# Increased Risk for ESBL-Producing Bacteria from Co-administration of Loperamide and Antimicrobial Drugs for Travelers’ Diarrhea^1^

**DOI:** 10.3201/eid2201.151272

**Published:** 2016-01

**Authors:** Anu Kantele, Sointu Mero, Juha Kirveskari, Tinja Lääveri

**Affiliations:** Medical Centre Aava, Helsinki (A. Kantele);; Karolinska Institutet, Solna, Stockholm, Sweden (A. Kantele);; University of Helsinki, Helsinki, Finland (A. Kantele, T. Lääveri);; Helsinki University Hospital (A. Kantele, T. Lääveri);; Helsinki University Hospital Laboratory, Helsinki (S. Mero, J. Kirveskari)

**Keywords:** loperamide, diarrhea, antidiarrheal, antibiotic resistance, travel medicine, travel, travelers’ diarrhea, antibiotics, antimicrobial drugs, extended-spectrum beta-lactamase–producing Enterobacteriaceae, ESBL, AMD, antimicrobial resistance, travelers’ diarrhea, travel health

## Abstract

Antimicrobial drug treatment of travelers’ diarrhea is known to increase the risk for colonization with extended-spectrum β-lactamase-producing *Enterobacteriaceae*. Among 288 travelers with travelers’ diarrhea, the colonization rate without medications was 21%. For treatment with loperamide only, the rate was 20%; with antimicrobial drugs alone, 40%; and with loperamide and antimicrobial drugs, 71%.

Resistance to antimicrobial drugs (AMDs) is predisposed in areas with poor hygiene and weak or nonexistent antimicrobial policy. Travelers visiting these areas presumably have a central role as transporters of multidrug-resistant intestinal bacteria across the globe (*1*), because a significant proportion of travelers (20%–70%) to high-prevalence areas become colonized with extended-spectrum β-lactamase-producing *Enterobacteriaceae* (ESBL-E) (*2*–*7*). Clinical infections do not develop in most travelers (*6*), and colonization is transient, waning within months (*7*). However, as evidenced by intrahousehold transmission from colonized patients after hospitalization, the bacteria may spread to household members (*8*,*9*) and eventually to local healthcare settings in the home countries of the travelers.

Several factors have been identified to increase the risk for ESBL-E colonization: travel destination (*2*–*7*), travelers’ diarrhea (TD) (*2*,*6*,*7*), use of AMDs (*5*–*7*), and age (*2*,*6*). In a recent study, we found that ESBL-E was contracted by 11% of travelers who did not have TD and did not take AMDs (TD–AMD–), 21% of those with TD who did not take AMDs (TD+AMD–), and 37% of those with TD who took AMDs (TD+AMD+) (*6*). Our conclusion that mild or moderate diarrhea should not be treated with AMDs raised questions about safe alternatives (*10*). In our previous study, probiotics appeared not to affect colonization (*6*). We found no studies that assessed possible risks posed by non-AMD antidiarrheal medications for treating TD, such as loperamide.

Loperamide, a drug with both antisecretory and antimotility effects (*11*), is widely used by travelers (*12*). Although mostly used alone, loperamide is sometimes used with AMDs; the combination stops symptoms faster than AMDs alone during the first 2 days of TD. After that, the combination no longer appears advantageous, probably because symptoms resolve naturally (*11*). Using loperamide with AMDs is presented as a safe option in general guidelines published by the US Centers for Disease Control and Prevention (*13*). However, the effects of co-administration on the risk for ESBL-E acquisition have not been addressed.

Some researchers have posed the question as to whether the antimotility effect of loperamide, involving prolonged passage through the gastrointestinal tract, would, in fact, increase the risk for colonization (data not shown). Such speculations prompted us to revisit our recent data (*6*) to compare loperamide, AMDs, and their combination in the treatment of TD with regard to the risk for contracting travel-acquired ESBL-E.

## The Study

We reviewed our recent data on ESBL-E acquisition among 430 travelers from Finland (*6*), selecting those with TD for separate analysis (Figure). All the volunteers provided fecal samples and completed questionnaires before and after travel. Symptoms of TD and use of medications, such as loperamide and AMDs, were included in the posttravel questionnaires. The countries visited were grouped as described (Table 1; *6*); processing of fecal specimens and identification of ESBL-E were detailed in our previous study (*6*). TD was defined by the World Health Organization criteria: passing >3 loose/liquid stools per 24 hours, or more frequently than normal (*14*).

**Figure:**
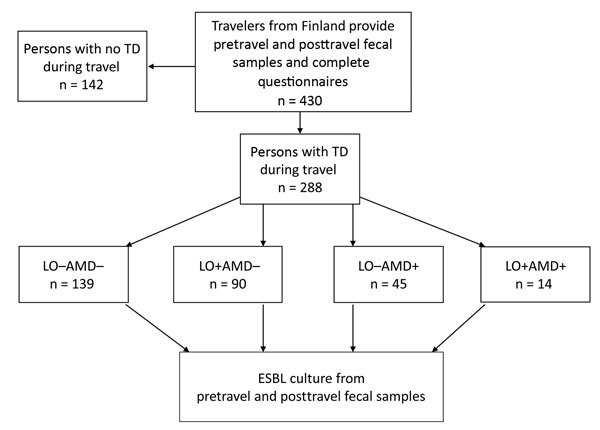
Study protocol for investigating risk for contracting ESBL-producing *Enterobacteriaceae* among travelers from Finland with TD. LO–AMD–, not treated with medication; LO+AMD–, treated with LO alone; LO–AMD+, treated with AMDs alone; LO+AMD+, treated with a combination of both drugs. AMD, antimicrobial drugs; ESBL, extended-spectrum β-lactamase; LO, loperamide; TD, travelers’ diarrhea.

**Table 1:** Characteristics of and co-administered treatments for 288 travelers with travelers’ diarrhea*

Characteristics	Total no. (%)	LO–AMD– no. (%)	LO+AMD– no. (%)	LO–AMD+ no. (%)	LO+AMD+ no. (%)
Total	288	139 (48)	90 (31)	45 (16)	14 (5)
Sex					
F	180 (62)	86 (62)	54 (60)	32 (71)	8 (57)
M	108 (38)	53 (38)	36 (40)	13 (29)	6 (43)
Age, y, median (IQR)	34 (25)	34 (26)	34 (23)	35 (24)	31 (38)
Geographic region					
South Asia	46 (16)	19 (14)	17 (19)	5 (11)	5 (36)
Southeast Asia	78 (27)	41 (29)	24 (27)	10 (22)	3 (21)
East Asia	4 (1)	1 (1)	1 (1)	2 (4)	0 (0)
Sub-Saharan Africa	130 (45)	62 (45)	39 (43)	23 (51)	6 (43)
North Africa and Middle East	5 (2)	3 (2)	1 (1)	1 (2)	0 (0)
South and Central America and the Caribbean	23 (8)	12 (9)	7 (8)	4 (9)	0 (0)
Europe and North America	2 (1)	1 (1)	1 (1)	0 (0)	0 (0)

*LO, loperamide; AMD, antimicrobial drugs.

Study participants were divided into 4 groups by treatment of TD: those taking no loperamide or AMDs (LO–AMD–); only loperamide (LO+AMD–); only AMDs (LO–AMD+); or loperamide plus AMDs (LO+AMD+). Those having taken AMDs for non-TD indications were categorized in groups with those with TD who took AMDs.

We used a multivariable binary logistic regression model to test our main hypotheses. Loperamide, AMDs, and their interaction (effect modification) were included in the model, along with risk factors that showed a p value <0.2 in univariate analysis in our previous study (*6*): sex, travel destination, use of AMDs, meals with residents of the location, contact with local healthcare, sites of meals, accommodations, duration of travel, age, and use of alcohol. Variables were eliminated to the final model by using backward selection of factors by Akaike Information Criteria, except for loperamide and the use of AMDs and their interaction, which were forced to the final model. Missing values were taken into account by multiple imputations, to reduce possible biases and efficiency loss, assuming that data were missing at random. We analyzed statistics using SPSS statistical software version 21 (IBM Corporation, Armonk, NY, USA).

Of all travelers in the previous study (*6*), a total of 288 of 430 (67%) who reported TD constituted the final study group (Table 1). ESBL-E was contracted by 26% of the subjects: 21% in the LO–AMD– group; 20% in the LO+AMD– group (adjusted odds ratio [aOR] 0.8, 95% CI 0.4–1.7); 40% in the AMD+LO– group (aOR 2.9, 95% CI 1.2–7.4); and 71% in the LO+AMD+ group (aOR 7.4, 95% CI 1.7–32.6) (Table 2). aOR for the interaction term of loperamide and AMDs was 3.1 (95% CI 0.6–16.6). Travel destination remained an independent risk factor, and sharing meals with locals appeared protective (Table 2). 

**Table 2:** Multivariable analysis of acquisition of extended-spectrum β-lactamase-producing *Enterobacteriaceae* by 288 travelers on the basis of administration of treatments for travelers’ diarrhea*†

Characteristics	Total, no. (%)	ESBL neg, no. (%)	ESBL pos, no. (%)	Univariate analysis			Multivariable analysis with imputation	
				p value	OR (95% CI)		p value	aOR (95% CI)
Total	288 (100)	213 (74)	75 (26)	NA	NA		NA	NA
Study groups								
LO–AMD–	139 (48)	110 (79)	29 (21)	NA	1.0		NA	1.0
LO+AMD–	90 (31)	72 (80)	18 (20)	0.874	0.9 (0.5–1.8)		0.583	0.8 (0.4–1.7)
LO–AMD+‡	45 (16)	27 (60)	18 (40)	0.012	2.5 (1.2–5.2)		0.022	2.9 (1.2–7.4)
LO+AMD+	14 (5)	4 (29)	10 (71)	<0.001	9.5 (2.8–32.4)		0.008	7.4 (1.7–32.6)§
Travel destination								
South Asia	46 (16)	21 (46)	25 (54)	NA	1.0		NA	1.0
Southeast Asia	78 (27)	48 (62)	30 (38)	0.087	0.5 (0.3–1.1)		0.186	0.6 (0.3–1.3)
East Asia	4 (1)	2 (50)	2 (50)	0.867	0.8 (0.1–6.5)		0.989	1.0 (0.1–12.3)
Sub-Saharan Africa	130 (45)	114 (88)	16 (12)	<0.001	0.1 (0.1–0.3)		<0.001	0.1 (0.05–0.3)
North Africa and Middle East	5 (2)	3 (60)	2 (40)	0.546	0.6 (0.1–6.7)		0.536	0.5 (0.1–3.8)
South and Central America and the Caribbean	23 (8)	23 (100)	0	NA	NA		NA	NA
Europe and North America	2 (1)	2 (100)	0	NA	NA		NA	NA
Other factors								
Sharing meals with locals¶	52 (19)	46 (88)	6 (12)	0.01	0.3 (0.1–0.8)		0.017	0.3 (0.1–0.8)
Contact with local healthcare	32 (11)	18 (56)	14 (44)	<0.001	2.5 (1.2–5.3)		0.314	1.7 (0.6–4.7)

*ESBL, extended-spectrum β-lactamase; OR, odds ratio; aOR, adjusted odds ratio; LO, loperamide; AMD, antimicrobial drugs; LO–AMD–,not treated with medication; LO+AMD–, treated with LO alone; LO–AMD+, treated with AMDs alone; LO+AMD+, treated with a combination of both drugs; NA, not applicable; pos, positive; neg, negative. †Values represent proportions with a given risk factor, aOR and p values in univariate and multivariable analysis. By using backward selection of factors by Akaike Information Criteria, the following factors were eliminated of the variables in the final model: age, duration of travel, sex, alcohol, site of meals, and type of accommodation. ‡Includes 7 travelers having taken antimicrobial drugs for indications other than TD. §aOR for interaction term of loperamide and AMDs is 3.1 (95% CI 0.6–16.6). ¶Information missing for 18 travelers.

Studies showing AMD treatment of patients with TD to be an independent risk factor for contracting ESBL-E (*5*–*7*) have evoked the question of less harmful treatments. The recommendation to restrict AMDs to severe cases (*5*,*6*,*15*) seems reasonable, as TD generally remains mild or moderate and resolves spontaneously (*12*,*15*). If symptoms require medical treatment, loperamide appears to be a sensible alternative for travelers who have no fever or bloody stools. However, because of its antimotility effect, its safety against contracting resistant intestinal bacteria has been questioned. Among studies that explored risk factors for ESBL carriage, we found none that showed data on the use of loperamide alone or in combination with AMDs.

Consistent with our previous analysis (*6*), we found AMD treatment of TD was an independent risk factor for colonization with ESBL-E; the rate increased from 21% (LO–AB–) to 40% (LO–AB+) (aOR 2.9, 95% CI 1.2–7.4). When used alone, loperamide did not add to the risk (20% colonized in the LO+AB– group).

In the group taking both loperamide and AMDs, the colonization rate was strikingly high, increasing from 21% (LO–AB–) to 71% (LO+AB+). The rate also appeared to exceed the risk for using AMDs alone (40%), yet the interaction term of loperamide and AMDs did not reach statistical significance (aOR 3.1, 95% CI 0.6–16.6), and the small subject number resulted in wide CIs. Theoretically, an additional increase in the risk seen in the combination group could be brought about by loperamide: because of its antimotility effect, contact time of the AMD to the gut lumen is increased, and the selection pressure posed by the AMD may be prolonged, thus intensifying its unfavorable effects. 

Our study design was limited by not including a randomized allocation of therapy and the varied use of loperamide according to symptoms. However, no association was seen between the severity of symptoms and acquisition of ESBL-E (data not shown).

Studies are needed to compare the relative risk posed by various AMD classes. Exploration of the influence of nonantimicrobial antidiarrheal agents with only antisecretory effect, such as racecadotril, as monotherapy and in combination with AMDs would also be beneficial. 

## Conclusions

Our results show that loperamide alone offers a safe alternative to AMDs for TD treatment because it does not add to the resulting in a low risk for acquiring drug-resistant intestinal bacteria. In contrast, combining loperamide with AMDs predisposes to ESBL-E colonization and may add to the substantial risk posed by AMDs alone. Our data dispute the safety of this combination.
